# The integrated common-sense model of illness self-regulation: predicting healthy eating, exercise behaviors, and health among individuals at risk of metabolic syndrome

**DOI:** 10.1186/s12889-023-16403-2

**Published:** 2023-08-04

**Authors:** Hui Zhang, Dandan Chen, Ping Zou, Jin Shao, Jingjie Wu, Nianqi Cui, Shuanglan Lin, Leiwen Tang, Qiong Zheng, Xiyi Wang, Zhihong Ye

**Affiliations:** 1 Department of Cardiology, Guizhou Provincial People’s Hospital, Guiyang, China; 2 Zhejiang University School of Medicine Sir Run Run Shaw Hospital, Hangzhou, China; 3 School of Nursing, Nipissing University, Toronto, M6J 3S3 Ontario Canada; 4 Department of Nursing, Zhejiang University School of Medicine, Hangzhou, China; 5 School of Nursing, Kunming Medical University, Kunming, China; 6 School of Nursing, The Hong Kong Polytechnic University, Hong Kong SAR, China; 7 School of Nursing, Shanghai JiaoTong University, Shanghai, China

**Keywords:** The common-sense model, Illness perception, Health behaviors, Metabolic syndrome

## Abstract

**Background:**

Little is known about the potential mechanisms of healthy eating and exercise change, and design interventions which aim to promote healthy eating and exercise change among individuals at risk of metabolic syndrome. This study aimed to identify key determinants of healthy eating, exercise behaviors, and health among individuals at risk of metabolic syndrome using the integrated common-sense model of illness self-regulation.

**Method:**

A cross-sectional study with a multi-wave data collection strategy. A total of 275 participants at risk of metabolic syndrome based on the clinical prediction model were included in the final analysis. Path analysis was employed to explore the pattern of relationships between key variables using AMOS.

**Results:**

The mediation analysis suggested that personal and treatment control, and coherence can positively affect self-reported health via intentions and health behaviors (exercise and healthy eating). Additionally, relationships between self-efficacy (exercise and healthy eating) and health outcomes can be mediated by health behaviors, and both intentions and health behaviors.

**Conclusions:**

This current research used the integrated common-sense model of illness self-regulation to predict healthy eating, exercise behaviors, and self-reported health among individuals at risk of metabolic syndrome. The results suggested that self-efficacy, intention, consequences, personal control, treatment control, and coherence were the key determinants of behavior and health, which can help design interventions to encourage healthy eating and exercise changes among individuals with a high risk of MetS.

## Background

Metabolic syndrome (MetS) is defined as a constellation of metabolic disorders, comprising abdominal obesity, hypertension, dyslipidemia, and insulin resistance [1]. It was estimated that 25% of the adult population suffers from MetS worldwide [2]. Individuals with MetS are more likely to develop coronary heart disease (CHD), other forms of cardiovascular atherosclerotic diseases (CVD), and diabetes mellitus type 2 (DMT2) [3]. Because of the high prevalence and poor outcomes of MetS, it has been considered a worldwide epidemic with significant morbidity, mortality and high socioeconomic cost [4].

According to the blueprint of Healthy China 2030, efforts should be made to identify risk factors of the disease and effectively prevent the disease with health promotion [5]. Clinical prediction models predict the risk of existing disease or future clinical outcomes among the target population using a combination of multiple risk factors (e.g., age, sex, and biomarkers). The early detection of individuals who are more likely to develop illness is of great importance using clinical prediction models, because it can allow for risk stratification. Healthcare professionals can estimate individual absolute risk of MetS in the future based on a prediction model that can help to design personalized care strategies. This means that high-risk patients can receive optimal care, such as therapeutic interventions, lifestyle changes, further diagnostic testing, or monitoring strategies, while preventing overtreatment in low-risk patients. This allows for optimal use of limited resources.

According to Transparent Reporting of a multivariable prediction model for Individual Prognosis or Diagnosis (TRIPOD), a systematic review was first conducted to evaluate all existing prediction models for MetS. The results suggested that existing prediction models for MetS had a high risk of bias in their methodological quality. Consequently, our team had developed and validated a prediction model for the 4-year risk of MetS in adults using logistic regression, which had satisfactory discrimination, calibration, brier score, and clinical utility [6–9]. After screening individuals with high risk of MetS using the above prediction model, it is important to help the target population change their healthy eating and exercise behaviors, which are also primary interventions for the management of MetS [10].

It is well known that theory-based interventions could help to interpret why and how the intervention components contribute to the overall effectiveness and success, while interventions without theoretical guidance often fail since potential explanatory mechanisms of the behavior change are missing, leading to ineffective translation of scientific evidence into knowledge and practice [11]. Therefore, an appropriate theoretical framework is crucial and recommended to help reveal the explanatory mechanisms of healthy eating and exercise changes, and design interventions which aim to encourage healthy eating and exercise changes among individuals with a high risk of MetS.

It is true that a message of high risk of MetS could serve as a stimuli for an individual in the form of a health threat. In response, people need to process and respond to information which signals a potential health threat to maintain everyday function and survival [12]. The common-sense model of illness self-regulation is a prominent social cognition approach, which outlines the dynamic processes where individuals perceive and respond to health threats, and relate to actions taken to cope with illness threats and illness-related information [13]. According to the common-sense model, individuals’ coping process for stimuli signaling health threats is guided by illness perceptions which are formed by information stored in memory relating to illnesses, and individuals’ cognitive processing of threat-related information [14]. Studies on the common-sense model have explored different dimensions of illness perceptions, including cause, consequences, identity, personal control, treatment control, illness coherence, emotion, and timeline [15].

Central to the common-sense model is that individuals’ different representations of illness perceptions could trigger the coping process to alleviate health threats and related distress. Consequently, coping strategies could have mediating effects on the relationships between the illness perceptions and outcomes in the model [14]. This means that illness perceptions could exert effects on adaptive and maladaptive outcomes by eliciting or suppressing coping strategies. The current evidence has summarized that threat perceptions (e.g., cause, consequences, identity, emotion) are more likely to be positively associated with avoidant coping strategies leading to maladaptive outcomes, including higher negative emotional responses, lower quality of life, lower likelihood of treatment-seeking behavior, and increased illness progression [12]. In contrast, dimensions in controllability of illness perceptions such as personal control, treatment control, and illness coherence, are found to be related to greater problem-focused coping strategies [12]. This facilitates adaptive outcomes, including better functioning, increased treatment seeking, reduced distress, and lower illness progression.

Although current findings have contributed to a better understanding of the explanatory mechanisms of coping initiated by illness perceptions based on the common-sense model, there are still some unsolved issues to which researchers have called for greater attention [12].

First, it is necessary to use behavior-specific measures as coping strategies instead of generalized coping procedures based on the common-sense model. Previous research usually employed generalized coping procedures, namely, avoidance/denial, cognitive reappraisal, expressing emotion, problem-focused coping strategies(generic), and seeking social support [16]. Compared to generic coping procedures, it was suggested that behavior-specific measures were more likely to contribute to stronger relationships between coping measures, illness perceptions, and outcomes [16]. Additionally, the principles of the common-sense model also state that this framework is more likely to predict behavior change (e.g., lifestyle changes) as coping strategies in response to health threats to improve health outcomes [14]. Consequently, it is needed to improve predictions in tests of the common-sense model by using behavioral coping strategies among individuals with high risk of MetS.

Second, previous studies indicated that emotion-focused coping strategies including emotion venting and denial can mediate the relationships between threat perceptions (e.g., consequences, identity, cause, timeline) and maladaptive outcomes [12]. However, few studies have demonstrated whether behavioral coping strategies mediated the association between illness perceptions and adaptive outcomes. According to the common-sense model, individuals could be motivated by illness perceptions to take action for managing the health threat, so it is necessary to test this fundamental prediction of the model among the population with high risk of MetS.

Third, if behavioral coping strategies is adopted, researchers are calling for integrating additional social cognition beliefs or constructs reflecting behaviors from other classic theories with the common-sense model to provide better and more accurate predictions for the relationships between beliefs, coping behaviors, and health outcomes [15–17]. Constructs from the theory of planned behavior can reflect beliefs about behaviors, and the central idea of the theory of planned behavior is that behavioral intention and perceived behavioral control can codetermine the performance of any behavior [18]. Among the constructs of the theory of planned behavior, intention presenting people’s plans of action has been proposed as the most proximal determinant of behavior change, which can mediate the relationships between beliefs and behaviors [18]. Additionally, perceived behavioral control refers to people’s confidence in their ability to perform a specific behavior and is regarded as a synonym for self-efficacy. According to a systematic review, integrating self-efficacy with the common-sense model can explain more proportion of the variance in behavior [17]. This means that self-efficacy is useful to understand health behavior change. Therefore, there is a need to integrate intention and self-efficacy from the theory of planned behavior with the common-sense model to better explain the antecedents of behavior change and health outcomes. It has been a trend to test intention and self-efficacy with illness perceptions in the common-sense model [19, 20].

**Fig. 1:**
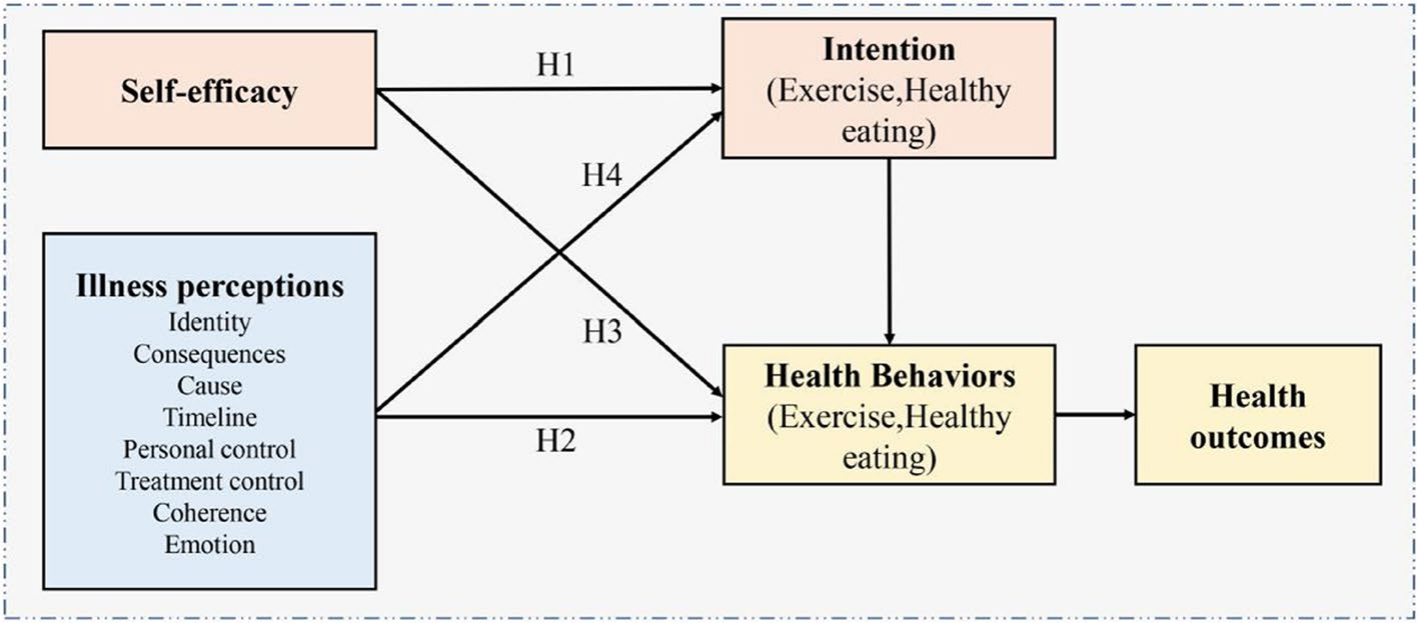
The integrated common-sense model

Based on the above, this study aimed to test an integrated common-sense model (Fig. 1) among individuals with a high risk of MetS to solve the key points in the current research about the common-sense model, increase its predictive validity, and provide a more comprehensive explanation of existing model tenets. As shown in Fig. 1, according to common-sense model, individuals’ different illness perceptions (e.g., identity, consequences, cause) could trigger behavior coping strategies to improve health outcomes (Hypothesis 1). Additionally, constructs from the theory of planned behavior were integrated with the common-sense model. Drawing on the theory of planned behavior, intention can mediate the relationships between self-efficacy and behaviors (Hypothesis 2). According to the integrated common-sense model, we also argued that self-efficacy could have indirect effects on health outcomes via health behaviors (Hypothesis 3). Illness perceptions can indirectly affect health outcomes through intention and health behaviors (Hypothesis 4).

## Methods

### Design and participants

This was a cross-sectional study with convenience sampling methods. Given the time sequence of the main variables based on the integrated theoretical model, a multi-wave data collection strategy was used to collect data, because it can avoid overlooking the temporal perspective of a mediational process for temporal order bias [21].

According to previous studies, three measurement periods, with a two-month time gap between each wave during the data collection process, were set for temporal separation between key variables from July 2021 to January 2022 [22, 23]. In the first wave (T1), illness perceptions, self-efficacy (healthy eating, exercise), and intention (healthy eating, exercise) were measured (the explanatory variable). In the second wave (T2, 2 months after the first wave), health behaviors (healthy eating, exercise) were measured (the mediator). In the third wave (T3, 2 months after the second wave), self-reported health was assessed (outcome variables).

Data were collected from the health promotion center of a tertiary care setting in Hangzhou. Participants were recruited according to the following inclusion criteria: (1) participants of at least 18 years of age or older, (2) participants were not diagnosed with metabolic syndrome (the diagnostic criteria was 2009 Joint Scientific Statement [24]) at the beginning of the present study, (3) individuals with high MetS risk. The exclusion criteria were: pregnant and breastfeeding women. Our team had developed a web-based calculator to predict the 4-year risk of developing MetS based on the prediction model [6–9], and the webpage calculator can be found at https://msypredict.shinyapps.io/dynnomapp/. Our previous work identified that age (years), total cholesterol (TC, mmol/l), serum uric acid (UA, μmol/l), alanine transaminase (ALT, U/L), and body mass index (BMI, Kg/m2) were identified as predictors for the prediction model. If the MetS risk was above 50.76 using the web-based calculator after inputting the predictor values, participants were included as having a high risk of developing MetS [25].

### Measures

#### Illness perceptions

The Brief Illness Perception Questionnaire (Brief IPQ) was used to measure illness perceptions. This is a 9-item scale measuring individuals’ cognitive and emotional representation of their illness or health threats [26]. Following the format of a previous study [27], each item started with “The problem with my health (a high risk of developing MetS)….” in our study. There was an open-ended item to assess causal perceptions where participants could list the three most likely causes of their illness or health threats in the Brief IPQ. A previous study suggested that there were seven main causal categories of illness perceptions: lifestyle, psychological causes, natural causes, working conditions, body changes, environmental factors, and other causes [28]. Therefore, these seven main causal categories of illness perceptions were provided in the open-ended item. The top three causes were chosen as a categorical variable. The other eight items are quantitative, and participants could rate each item from 0 to 10: (1) consequences; (2) timeline; (3) personal control; (4) treatment control; (5) identity; (6) concern; (7) coherence; and (8) emotional response. Concern and emotional response can be merged to form an item named emotional representations [26]. Higher total scores represent a worse degree of perceived health threats. Test–retest reliability, discriminant validity and concurrent validity of the Brief IPQ have been previously proven [29].

#### Self-efficacy

Self-Rated Abilities for Health Practices Scale is a five-point scale ranging from 0 (not at all) to 4 (completely), and it was used to assess participants’ self-efficacy to implement health-promoting behaviors (Exercise, Nutrition, Responsible Health Practice, and Psychological Well Being) [30]. In the present study, two subscales (7 items for exercise, 7 items for nutrition) were employed. Higher scores mean greater self-efficacy for health behaviors (exercise, healthy eating). The Cronbach’s α was 0.89 and 0.75 for exercise and nutrition, respectively.

#### Healthy eating intention

A healthy eating intention scale was developed by Kara Chan [31]. Two questions on a 5-point scale ranging from 1 (definitely no) to 5 (definitely yes) were used to measure healthy eating intention (e.g., “Do you intend to engage in healthy eating over the next week?” and “How likely is it that you will engage in healthy eating over the next week?”). The Cronbach’s alpha coefficient was 0.90.

#### Exercise intention

An exercise intention scale was developed by Ming Fang [32]. Two questions on a 5-point scale ranging from1 (definitely no) to 5 (definitely yes) were used to measure exercise intention (e.g., “Do you intend to do exercise in the next two months? ", "Do you plan to do exercise in the next two months?", and "Are you looking forward to exercise in the next two months?”). The Cronbach’s alpha coefficient was 0.96.

#### Health behaviors

Health behaviors were measured using the Health-Promoting Lifestyle Profile II (HPLP-II) to assess the frequency of self-reported health-promoting behaviors in the domains of health responsibility, physical activity, nutrition, spiritual growth, interpersonal relations and stress management [33]. Nine items for nutrition and eight items for physical activity were used in this study. The scale is a 4-point Likert scale ranging from 1 (never) to 4 (routinely). The Cronbach’s alpha coefficients were 0.75 and 0.78 for nutrition and physical activity, respectively.

#### Self-reported health

One question was used to measure self-reported health. Participants were asked “How would you rate your health status?” [34]. In the scale, 0 means "the worst possible health" and 10 means "the best possible health [35].

#### Data analysis

Descriptive statistics and Spearman’s correlations were used to assess the relationships between illness perceptions, self-efficacy, healthy eating, exercise, and self-reported health. Reported p-values are based on 2-sided tests in this study. According to the integrated common-sense model of illness self-regulation, path analysis was employed to explore the pattern of relationships between key variables by using AMOS. Path analysis can examine how well a hypothesized structural model fits the collected data among the included constructs [36]. The following goodness-of-fit indices in SEM were chosen to evaluate the adequacy of the model: chi-square statistic (*P* > 0.05), the Root Mean Square Error of Approximation (RMSEA < 0.08), Goodness-of-Fit Index (GFI > 0.9), Adjusted Goodness-of-Fit Index (AGFI > 0.9), Comparative Fit Index (CFI > 0.9), Tucker-Lewis Index (TLI > 0.9), and chi-square divided by the degrees of freedom (χ^2^ /df < 2). The model was estimated separately for healthy eating and exercise, respectively. The SEM and the bootstrap method (5,000 replicates) [37] were employed to examine the mediation effects of illness perceptions and self-efficacy on health behavior, intention, and self-reported health. The mediation effects are identified when zero is not contained in the bootstrap 95% confidence interval.

## Results

### Sociodemographic characteristics of participants

**Fig. 2:**
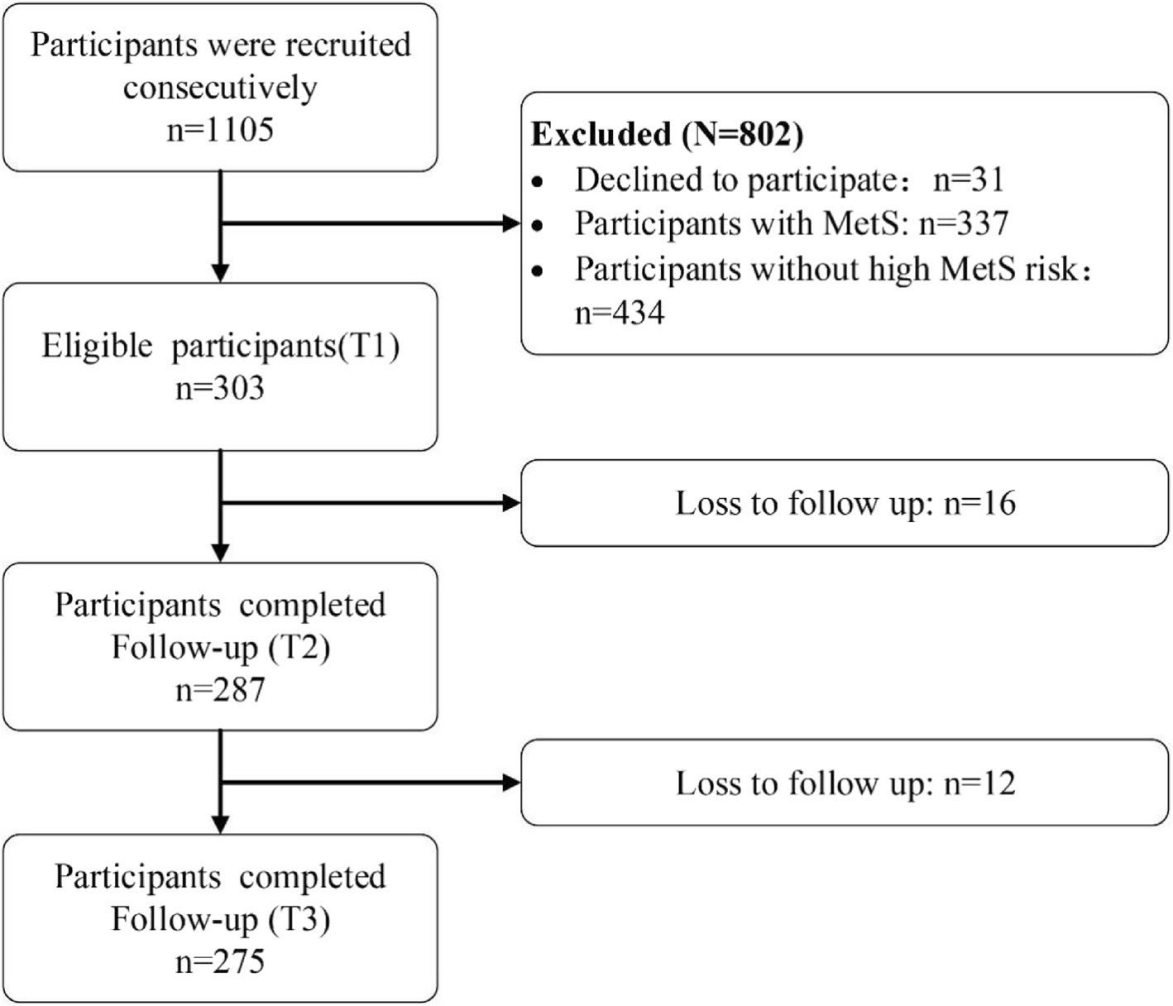
The flowchart of recruitment and follow-up

A total of 1,105 participants were sampled, and 275 participants were included in the final analysis (Fig. 2). The mean age was 53.93 (SD = 7.985) years. The majority of the final sample were men (71.64%) and Han Chinese (97.45%). More than 50% of participants had no religion. 94.18% of participants were married. The sociodemographic characteristics of the participants is shown in Table 1.

**Table 1:** Descriptive statistics of the characteristics of the sample (*n* = 275)

Variables	n (%)/Mean ± SD
**Age**	53.93 ± 7.985
**Sex**	
Female	78 (28.36%)
Male	197 (71.64%)
**Nationality**	
Han	268 (97.45%)
Others	7 (2.55%)
**Marital status**	
Single	7 (2.55%)
Married	259 (94.18%)
Divorced	6 (2.18%)
Widowed	3 (1.09%)
**Religion**	
No	184 (66.91%)
Buddhism	78 (28.36%)
Christianity	8 (2.91%)
Others	5 (1.82%)
**Education**	
Elementary school or under	27 (9.82%)
Middle school	73 (26.55%)
High school education or technical secondary school	52 (18.91%)
Junior college	42 (15.27%)
University education or above	81 (29.45%)
**Average household monthly income (Yuan)**	
< 5,000	18 (6.55%)
5,000–10,000	43 (15.64%)
10,000–20,000	90 (32.73%)
> 20,000	124 (45.09%)
**Smoking**	
Yes	77 (28.00%)
No	198 (72.00%)
**Drinking**	
Yes	143 (52.00%)
No	132 (48.00%)

### Correlations among main variables

The results of correlational analyses among the main variables are presented in Table 2. The findings suggested that self-reported health was positively correlated with exercise, exercise intention, exercise self-efficacy, healthy eating, healthy eating intention, healthy eating self-efficacy, treatment control, symptoms, and coherence. Exercise was positively correlated with exercise intention, exercise self-efficacy, consequences, personal control, identity, treatment control, and coherence. Healthy eating was positively correlated with healthy eating intention, healthy eating self-efficacy, personal control, treatment control, and coherence.

**Table 2:** Correlations among primary variables (*n* = 275)

Variables	Mean	SD	1	2	3	4	5	6	7	8	9	10	11	12	13	14	15
1 Self-reported health	6.749	1.223	1														
2 Exercise	2.319	0.501	0.351**	1													
3 Exercise intention	3.474	1.045	0.349**	0.750**	1												
4 Exercise self-efficacy	2.807	0.674	0.311**	0.701**	0.648**	1											
5 Healthy eating	3.124	0.390	0.578**	0.398**	0.324**	0.336**	1										
6 Healthy eating intention	3.802	0.835	0.375**	0.482**	0.681**	0.379**	0.477**	1									
7 Healthy eating self-efficacy	3.026	0.525	0.380**	0.462**	0.439**	0.611**	0.488**	0.369**	1								
8 Consequences	2.164	2.115	0.019	0.134*	0.124*	-0.047	0.023	0.185**	-0.019	1							
9 Timeline	4.276	1.962	-0.017	-0.056	-0.018	-0.089	-0.027	-0.027	-0.115	0.291**	1						
10 Personal control	5.971	1.526	0.305**	0.583**	0.620**	0.436**	0.412**	0.580**	0.366**	0.214**	-0.105	1					
11 Treatment control	7.189	1.460	0.323**	0.475**	0.590**	0.339**	0.346**	0.634**	0.300**	0.148*	0.033	0.699**	1				
12 Identity	0.756	1.206	0.070	0.166**	0.129*	0.083	0.081	0.11	0.117	0.406**	0.173**	0.220**	0.144*	1			
13 Coherence	4.113	2.284	0.262**	0.426**	0.548**	0.361**	0.269**	0.485**	0.221**	0.218**	0.153*	0.528**	0.568**	0.336**	1		
14 Emotional representations	0.502	0.799	-0.074	0.004	-0.027	-0.085	-0.072	0.020	-0.047	0.241**	0.067	0.024	0.042	0.233**	0.005	1	
15 Causes	0.596	0.492	-0.035	-0.031	-0.041	-0.059	-0.056	0.022	-0.084	-0.036	0.004	0.002	0.022	-0.037	-0.030	0.048	1

Note: ** *P* < 0.01 * *P* < 0.05

#### The mediation analysis

**Fig. 3:**
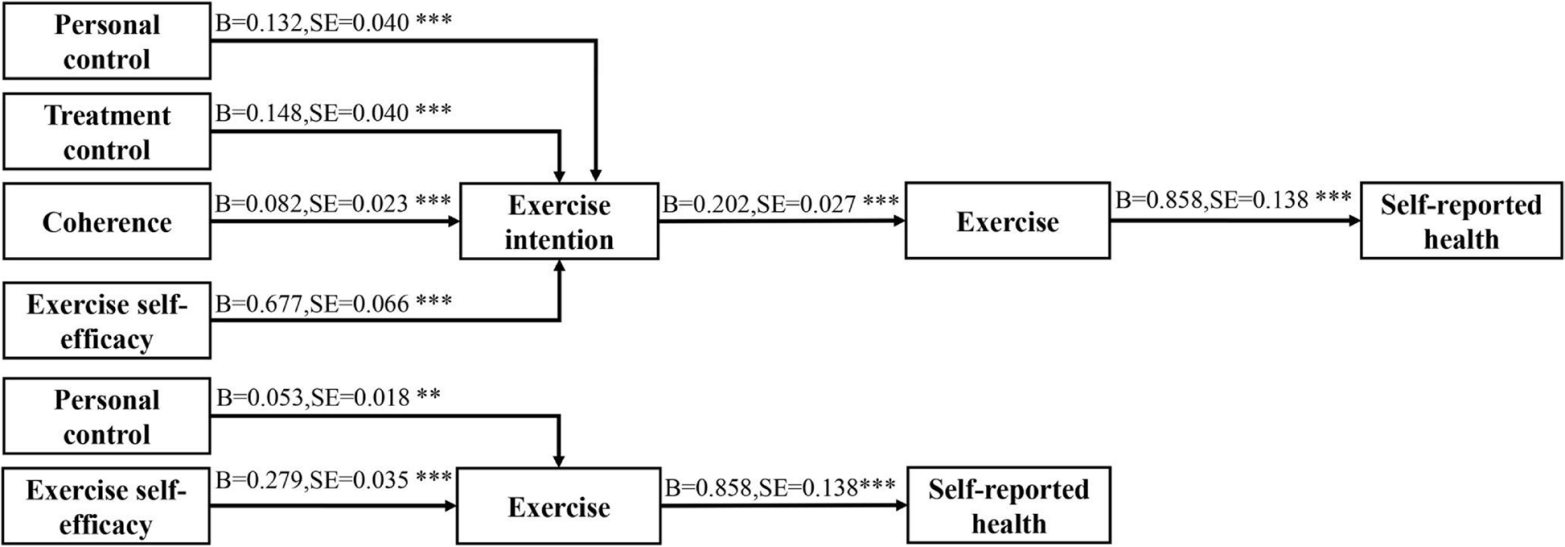
The significant indirect effects (Exercise)

The hypothesized model for exercise had satisfactory fit indices: *P* > 0.05, RMSEA = 0.045, GFI = 0.991, AGFI = 0.929, CFI = 0.995, TLI = 0.967, and χ^2^ /df = 1.548. As shown in Table 3 and Fig. 3, the results of mediation analysis suggested that there were several significant indirect paths: personal control → exercise intention → exercise → self-reported health, treatment control → exercise intention → exercise → self-reported health, coherence → exercise intention → exercise → self-reported health, exercise self-efficacy → exercise intention → exercise → self-reported health, personal control → exercise → self-reported health, and exercise self-efficacy → exercise → self-reported health.

**Table 3:** The mediation analyses for exercise (*n* = 275)

**Variables**	Point estimate	Product of coefficients		Bootstrapping	
				Bias-corrected 95% CI	
		SE	Z	Lower	Upper
Consequences → exercise intention → exercise → self-reported health	0.005	0.004	1.250	-0.002	0.014
Timeline → exercise intention → exercise → self-reported health	0.000	0.004	0.000	-0.007	0.008
**Personal control → exercise intention → exercise → self-reported health**	**0.023**	**0.009**	**2.556**	**0.008**	**0.045**
**Treatment control → exercise intention → exercise → self-reported health**	**0.026**	**0.009**	**2.889**	**0.011**	**0.048**
Identity → exercise intention → exercise → self-reported health	-0.010	0.008	-1.250	-0.027	0.004
**Coherence → exercise intention → exercise → self-reported health**	**0.014**	**0.005**	**2.800**	**0.006**	**0.028**
Emotional representation → exercise intention → exercise → self-reported health	0.000	0.010	0.000	-0.023	0.019
Cuases → exercise intention → exercise → self-reported health	-0.006	0.014	-0.429	-0.033	0.021
**Exercise self-efficacy → exercise intention → exercise → self-reported health**	**0.117**	**0.026**	**4.500**	**0.076**	**0.179**
Consequences → exercise → self-reported health	0.012	0.010	1.200	-0.007	0.033
Timeline → exercise → self-reported health	-0.003	0.008	-0.375	-0.020	0.011
**Personal control → exercise → self-reported health**	**0.046**	**0.018**	**2.556**	**0.013**	**0.086**
Treatment control → exercise → self-reported health	-0.001	0.014	-0.071	-0.030	0.027
Identity → exercise → self-reported health	0.012	0.014	0.857	-0.012	0.044
Coherence → exercise → self-reported health	-0.009	0.010	-0.900	-0.029	0.011
Emotional representation → exercise → self-reported health	0.012	0.020	0.600	-0.024	0.055
Cuases → exercise → self-reported health	0.005	0.031	0.161	-0.059	0.064
**Exercise self-efficacy → exercise → self-reported health**	**0.240**	**0.046**	**5.217**	**0.158**	**0.341**

**Fig. 4:**
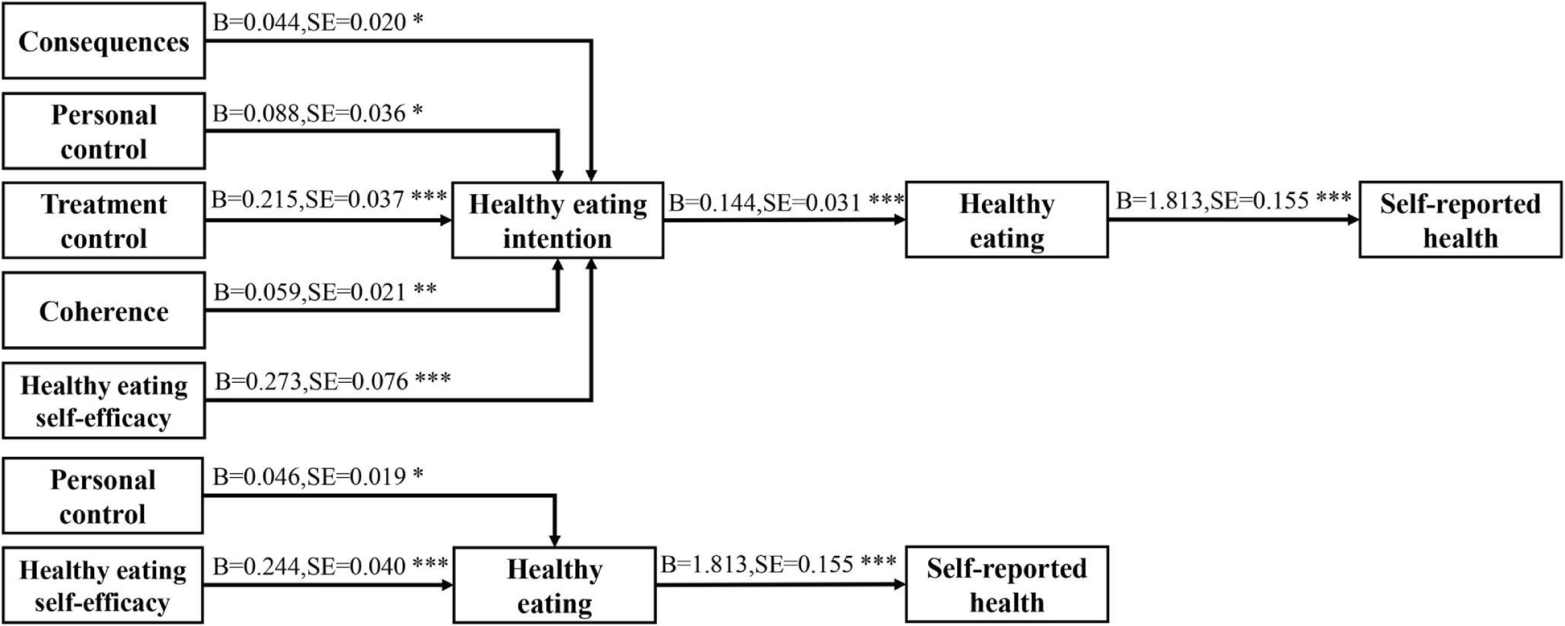
The significant indirect effects (Healthy eating)

The hypothesized model for healthy eating had satisfactory fit indices: *P* > 0.05, RMSEA = 0.037, GFI = 0.992, AGFI = 0.937, CFI = 0.996, TLI = 0.972, and χ^2^ /df = 1.376. As shown in Table 4 and Fig. 4, the results of mediation analysis suggested that there were several significant indirect paths: consequences → healthy eating intention → healthy eating → self-reported health, personal control → healthy eating intention → healthy eating → self-reported health, treatment control → healthy eating intention → healthy eating → self-reported health, coherence → healthy eating intention → healthy eating → self-reported health, healthy eating self-efficacy → healthy eating intention → healthy eating → self-reported health, personal control → healthy eating → self-reported health, and healthy eating self-efficacy → healthy eating → self-reported health.

**Table 4:** The mediation analyses for healthy eating (*n* = 275)

**variables**	Point estimate	Product of coefficients		Boststrapping	
				Bias-corrected 95% CI	
		SE	Z	Lower	Upper
**Consequences → healthy eating intention → healthy eating → self-reported health**	**0.012**	**0.006**	**2.000**	**0.002**	**0.026**
Timeline → healthy eating intention → healthy eating → self-reported health	-0.005	0.005	-1.000	-0.018	0.004
**Personal control → healthy eating intention → healthy eating → self-reported health**	**0.023**	**0.011**	**2.091**	**0.006**	**0.048**
**Treatment control → healthy eating intention → healthy eating → self-reported health**	**0.056**	**0.017**	**3.294**	**0.029**	**0.097**
Identity → healthy eating intention → healthy eating → self-reported health	-0.017	0.010	-1.700	-0.040	0.001
**Coherence → healthy eating intention → healthy eating → self-reported health**	**0.015**	**0.007**	**2.143**	**0.005**	**0.033**
Emotional representation → healthy eating intention → healthy eating → self-reported health	0.001	0.013	0.077	-0.026	0.028
Causes → healthy eating intention → healthy eating → self-reported health	0.011	0.021	0.524	-0.027	0.055
**Healthy eating self-efficacy → healthy eating intention → healthy eating → self-reported health**	**0.071**	**0.025**	**2.840**	**0.033**	**0.136**
Consequences → healthy eating → self-reported health	-0.021	0.018	-1.167	-0.060	0.014
Timeline → healthy eating → self-reported health	0.022	0.021	1.048	-0.017	0.066
**Personal control → healthy eating → self-reported health**	**0.084**	**0.042**	**2.000**	**0.005**	**0.170**
Treatment control → healthy eating → self-reported health	-0.027	0.041	-0.659	-0.105	0.055
Identity → healthy eating → self-reported health	0.004	0.032	0.125	-0.068	0.058
Coherence → healthy eating → self-reported health	-0.005	0.022	-0.227	-0.048	0.038
Emotional representation → healthy eating → self-reported health	-0.048	0.042	-1.143	-0.132	0.032
Causes → healthy eating → self-reported health	-0.052	0.071	-0.732	-0.197	0.086
**Healthy eating self-efficacy → healthy eating → self-reported health**	**0.441**	**0.083**	**5.313**	**0.287**	**0.619**

## Discussion

This is the first study to examine the associations between self-efficacy, intention, illness perceptions, health behaviors (exercise and healthy eating), and self-reported health by using a multi-wave data collection strategy among individuals with a high risk of MetS. Based on an integrated theoretical model derived from two prominent social cognition theories, we proposed that illness perceptions and self-efficacy can not only be regarded as predictors of intention to participate in health behaviors contributing to health outcomes, but they can also improve health outcomes via health behavior change only. Three waves of data were collected to ensure that mediators were measured prior to the outcome, and two models were tested which exhibited adequate fit with the data according to the goodness-of-fit indices. The findings suggested that the hypotheses were partially supported.

The indirect effects of consequences on self-reported health were found to be significant through both healthy eating intention and healthy eating among the population with a high risk of MetS. This pattern is consistent with the proposal of the common-sense model, which suggested that threat representations can motivate coping behaviors leading to adaptive outcomes. Additionally, empirical evidence also supported this pattern. For example, Brewer et al. found that higher levels of medication adherence mediated the associations between cholesterol levels and perceived consequences of hypercholesterolemia [38]. The findings of the present study illustrated that individuals could be guided by a high risk of MetS to initiate healthy eating for better health outcomes. Previous studies revealed that threat perceptions had a positive indirect effect on maladaptive outcomes via emotion-focused coping procedures, while our study has suggested that the relationships between threat perceptions and adaptive outcomes could be mediated by specific coping behaviors. This means that illness representations signaling threat (e.g., identity, consequences, and timeline) may have two sets of specific indirect effects on adaptive or maladaptive outcomes through emotion-focused coping procedures and specific coping behaviors.

In the present study, we found that personal control was indirectly associated with self-reported health through behavioral coping (exercise and healthy eating). Additionally, the results of serial multiple mediation analysis suggested that there were several significant indirect paths. Current findings suggested that perceptions representing personal capacity to manage the health threats, including personal and treatment control and coherence perceptions representing perceived clarity in understanding illness, can positively affect self-reported health via intentions and health behaviors (exercise and healthy eating). The indirect effects of personal control on self-reported health mediated through behavioral coping (exercise and healthy eating) in the present models are consistent with the theory and prior research [12]. The relationships between perceptions reflecting less threat and self-reported health were serially mediated by intentions and health behaviors (exercise and healthy eating). The reason to explain this pattern of effects may be that the tendency for individuals to adopt behavioral coping strategies is adaptive (i.e., forming intentions to do something about the illness) if they believe that personal capacity and illness understanding make the risk message less threatening, and health behaviors can alter the course of the illness and improve health outcomes [12]. Previous research used generic coping procedures rather than relying on behavior-specific measures as coping responses [15]. In contrast, we found that coping behavior could be more likely to capture means to cope with health threats due to closer correspondence with the perceptions and health outcomes. Moreover, motivation toward behavior coping can be triggered by intentions which have been identified as the most proximal and effective predictor of behavior in health contexts and are of extreme importance to understanding behavior because they reflect a strong commitment by the individual to engage in specific behavior [39], the findings also suggested that intentions were sufficient to be mediators in the relationships between beliefs about illness, coping behaviors (exercise and healthy eating), and health outcomes.

While a high risk of MetS is related to an increased risk of chronic illness, intentions and health behaviors did not mediate the associations between the threat perceptions (e.g., cause, consequences, identity, emotion) and health outcomes. The reason may be that MetS patients with comorbid conditions (e.g., CHD, CVD, DMT2) are likely to have elevated anxiety and distress, but individuals with a high risk of MetS believe that the current condition is asymptomatic and there is no indication of future illness and treatment, so it was likely that threat perceptions will be of insufficient strength to form behavioral intentions. Another reason could be explained by the Chinese cultural and ethnic backgrounds. One of the key concepts in Chinese thought and culture is called “The Middle Way”, which refers to people complying with the middle way in both speech and action. This means that although individuals realized that the high risks of developing MetS could be harmful to their health, keeping calm and taking no action can be seen as the appropriate way to cope with the problem in the current situation.

The other primary findings of this study were that the relationships between self-efficacy (exercise and healthy eating) and health outcomes can be mediated by health behaviors (simple mediations) and both intentions and health behaviors (serial multiple mediations). This was consistent with previous research, which suggested that higher levels of self-efficacy were associated with more engagement in health behaviors, and self-efficacy can be theorized to be a determinant of intention contributing to the importance of self-efficacy [40, 41]. Hagger et al. have also stated that as behavioral coping can be regarded as coping responses, incorporating constructs (e.g., self-efficacy and intentions) from social–cognitive theories in the common-sense model can be accounted for by identifying the determinants of health behaviors and health outcomes [15]. We found that self-efficacy was a stronger predictor of intentions, exercise, and healthy eating than other constructs of illness perceptions, and this was also consistent with prior research which found that self-efficacy was more important than perceptions of risk in health contexts [42]. Moreover, it is not only self-efficacy that can indirectly affect health behaviors but self-efficacy can also indirectly affect health outcomes. This is because self-efficacy can influence behavior by affecting cognitive processes (e.g. planning for behavior), motivational processes (e.g. improved commitment to goals), and regulating potentially disruptive affective processes (e.g. fear of failure), contributing to better outcomes eventually. For example, if individuals feel confident in their ability to participate in exercise, attending an exercise class is an enactive mastery experience that can lead to better health conditions.

### Implication

Since health behaviors are important to improve individuals’ wellbeing, efforts should be made to understand and attempt to change the behavioral processes contributing to behavioral change interventions. The theories could help to identify key constructs associated with behavioral change which might be targeted as the mechanisms of interventions. Consequently, research can applicate social cognition theories to identify the determinants of health-related behaviors, forming evidence for behavioral interventions [20, 40].

This present study shed light on the integrated common-sense model of illness self-regulation to offer the unique and complementary information needed in developing interventions among individuals with a high risk of MetS based on key determinants. The results revealed the mechanisms of action in designing interventions for exercise and healthy eating, including self-efficacy, consequences, personal control, treatment control, and coherence which were proposed to be the key components of the intervention. Behavior change techniques are defined as an observable, active, and replicable component of an intervention based on potential mechanisms of action, which can be designed to alter the processes of regulating behavior [43]. It is suggested that effective behavior change techniques have been used to develop interventions to increase exercise and healthy eating [44]. For example, there are four behavior change techniques for enhancing self-efficacy when developing interventions, such as mental rehearsal of successful performance, self-talk, focus on past success, and verbal persuasion [44]. Additionally, clinical practitioners could also use the MAP model as a practical tool for selecting behavior change techniques. The reliable grouping structures of the MAP model including motivation, action, and prompts can help practitioners and researchers select related and effective behavior change techniques in a given context [45].

### Limitations

There were some limitations restricting the generalizability of the current findings. First, this research adopted a correlational, cross-sectional design, so the causal direction of the proposed effects cannot be determined. Second, we only integrated two important constructs from another social cognition theory. It is crucial to consider more appropriate constructs to provide a comprehensive explanation of existing model tenets. Third, the integrated model was only examined in specific cultural and ethnic backgrounds, so samples of various cultural or ethnic backgrounds are needed to draw generalizable conclusions. Lastly, intentions and health behaviors did not mediate the associations between the illness perceptions and health outcomes in our study, because participants were asymptomatic and may have considered the health threats of insufficient strength to trigger intentions and behaviors. Future studies can include participants with risk of illness who may already have symptoms to test the role of intentions and health behaviors within the integrated common-sense model.

## Conclusion

This current research used the integrated common-sense model of illness self-regulation to predict healthy eating, exercise behaviors, and self-reported health among individuals at MetS risk. The results suggested that self-efficacy, intention, consequences, personal control, treatment control, and coherence were the key determinants of behavior and health. The findings can help healthcare professionals choose behavior change techniques to design interventions to change self-efficacy, consequences, personal control, treatment control, and coherence improving health behaviors.

## Data Availability

The data is available, upon a reasonable request, from the corresponding author.

## Abbreviation

MetS: Metabolic syndrome
